# Normal Ovariotomy

**Published:** 1873-05

**Authors:** Robert Battey

**Affiliations:** Rome, Georgia


ATLANTA

### MEDICAL AND SURGICAL JOURNAL.

VOL. XI.—MAY, 1873.—NO. 2.

---

### ORIGINAL COMMUNICATIONS.

---

### NORMAL OVARIOTOMY.

BY ROBERT BATTEY, M.D., ROME, GEORGIA.

*Read Before the Georgia Medical Association, Atlanta, Georgia, April,* 1873.

[CONTINUED FROM LAST MONTH.]

It has been generally supposed that the removal of the ovaries would be attended, necessarily, with *loss of the aphrodisiac sense.* This, however, appears not to be the result, certainly not the uniform result.

In the case of Miss K. V., of Baltimore, previously cited upon another point, and in which both ovaries were removed, Dr. Atlee remarks: "She married, made a visit to Europe, and after her return I learned through her mother that *the sexual feelings were normal.*"

Upon this point Dr. Peaslee* says: "Thus double ovariotomy, as a rule, is not followed by any loss of the special characteristics of woman; the only decided physiological change being a final cessation of menstruation as well as ovulation. Three of my own patients, married and highly educated ladies, after recovery again became splendid examples of womanhood, enjoying the most perfect health, and retaining all their former

* Medical and Surgical Treatment of Women. p. 160.

attributes of mind as well as of body, and *with undiminished sensory capacities in their matrimonial relations.*"

On the 18th of March, 1871, Dr. Meadows,* in the Hospital for Women, London, removed both ovaries, the patient recovering without a bad symptom. She was seen again on 1st August, when "she stated that she continued to menstruate quite regularly, and *has sexual desire as much as ever.*"

Mr. Spencer Wells† mentions a case of double ovariotomy, in his practice, where the patient, after recovery, not only retained her sexual powers, but in an exaggerated degree, and "*actually became aggressive in her demeanor.*"

My friend, Prof. John T. Darby, of South Carolina, informs me, in a private letter, that he is assured by a patient from whom he removed the uterus and both ovaries, that she enjoys the approaches of her husband as much as ever.

The *loss of the menstrual molimen* is the condition of cure, it is the rolling off of a *burden*; in no sense can it be considered a calamity.

Of the *loss of outward feminine graces*—of mammary atrophy, change of voice, growth of beard, etc., it may be said that such occurrences after double ovariotomy are exceptional, and not the rule. An observation of Dr. Peaslee upon this point has already been quoted, and he further remarks: "It has also been asserted that women from whom both ovaries have been removed undergo a decided physiological change, they becoming thin, their features more masculine, the voice harsh, the breasts atrophied, and sometimes a well-developed beard resulting from the operation. In Mr. Potts' case, already cited, the first-mentioned two of the preceding changes occurred; in a case reported by Dr. A. Reeves Jackson, the voice became harsher and more masculine, but otherwise no change was discernible; and Dr. W. L. Atlee found one of his patients, after double ovariotomy, with a "shaved beard." This was, however, fourteen years after the operation; and the change probably occurred during the previous three years, the patient passing from forty-seven to fifty years of age, since Dr. Atlee makes no mention of the change as having occurred when he visited her at the age of forty-seven. I have seen a well-developed beard in three in-

* London Lancet. Reprint June, 1872. p. 324.
† American Journal of Obstetrics, May, 1872. p. 131.

stances in women who had cystic disease of the ovary, and should accept such a fact as a ground of suspicion that both ovaries are affected.

"But on the other hand, it should be stated that the same changes which have been mentioned above have been observed in women, especially when somewhat advanced in life, who have never been suspected of ovarian disease of any form.

"The cases I have just detailed must also be regarded as exceptional. In three of my first six cases, both ovaries were removed, and neither of the patients has lost any of her feminine attributes up to the present time, so far as indicated by external appearances. The time which has elapsed since the three operations is twenty-one, sixteen and nine years. I have since had six successful cases of double ovariotomy, in neither of which has any change in the physical appearance occurred; but the time with these has only been from six to two years. Nor do Dr. Clay and Mr. Wells, with their extensive experience, mention any instances of the occurrence of such changes.

"It can not, therefore, be stated, as a general proposition, that the removal of the ovaries produce a change in the physical organization of woman, so as to make her more masculine in appearance, voice and form; though such changes sometimes follow double ovariotomy, as well as attend the progress of ovarian disease in cases not submitted to that operation, but they also occur independently of any suspected ovarian lesion."

Do I hear some one exclaim with indignant surprise, "What! Ovariotomy for amenorrhœa!"

Ovariotomy for amenorrhœa? *By no means!* I beg not to be so misunderstood; I make no such proposition. When I reflect that the menopause is but a sort of physiological amenorrhœa, so to speak, how could I propose to cure one amenorrhœa by substituting another amenorrhœa? I trust I shall not be accused of the heresy *similia similibus, etc.* What I do propose is this: *Ovariotomy to determine the change of life; and the change of life for* ANY GRAVE DISEASE *which is incurable without it, and which is curable with it.*

But it may be asked, What necessity is there for surgical interference in these cases? I answer, it is necessary because the pathological conditions for which the remedy is proposed are: *First, destruction of human life; second, destruction of human*

*health; third, destruction of human reason; fourth, destruction of human happiness; and fifth, incurable by the recognized resources of our art.*

Who that has stood by the bedside of his unconscious patient and witnessed the distended veins, the throbbing arteries, the suffused, darkened countenance, and perchance the violent contortions of obstinately recurring convulsions, and has not felt that the human organism before him was engaged in a fearful conflict? Can it be that the brain, the lungs, the heart, and other vital organs, stand by as idle spectators while the battle fiercely rages? Nay, are not the heart, the brain, and the spinal marrow already actively engaged in the contest? and shall they all, and always, come off the field unscathed?

When we consider for a moment the intricate system of telegraphic intercommunication by which nature connects all the various forces of her grand army with one composite whole, and by which she rallies one, or all, at her pleasure, upon the point of attack, is it a wonder that the brain, the heart, the lungs, or, indeed, any other organ, should suffer irreparable damage?

If, after many such conflicts, we should find apoplexy, cerebral softening or mania, need we be surprised? If, under the stress of labor it has to undergo, the heart becomes dilated or hypertrophied; if the endocardium or pericardium inflame; if the valves break down, and are no longer able to perform their appointed office, are we filled with wonder? If we find the lungs suffering pneumonia, pulmonary apoplexy, or tubercular deposit, do we raise our hands in astonishment?

Suppose we find a patient whose history shows these repeated vascular conflicts—as for instance my first cited case, Miss Mary M.—must we search industriously for a long-forgotten rheumatic pain in some distant joint to explain her broken-down heart? But we have not time to comment at length upon this subject. I shall read you brief statements of a few cases, and hurry onward.

Before the Obstetrical Society of Philadelphia, Dr. R. G. Curtin read the following history: C. W., a stout, robust, middle-aged Irish woman, was admitted August 28th, 1866, to the insane department of the Philadelphia Hospital, laboring under an attack of acute mania, brought on by intemperance. After her admission, in a lucid interval, she informed Dr. Curtin that she had *never menstruated*, but that periodically she had pains in the head and back, with all the other symptoms usually accompanying the performance of this function, and *attributed her mental condition, in a measure, to the absence of the flow.* She had been married and living with her husband for ten years, and had *never conceived.* The maniacal symptoms returned, and she *died, very suddenly*, three days after her admission.

"On post-mortem examination no abnormal condition of the brain could be discovered; the immediate cause of death was *an extensive pulmonary apoplexy.* The external sexual organs were normal, the uterns undersized, but with all its appendages complete. On both fallopian tubes, about midway between their extremities, were beard-like prominences. On laying them open two calcareous nodules were found, each about the size of a pea, and completely obstructing the calibre of the tubes, which, above these points, were dilated and distended by fluid. *The ovaries were marked with numerous cicatrices of ruptured Graafian vesicles, one apparently being only three weeks old.* No evidences of pelvic peritonitis.

"Dr. Wm. Pepper remarked that cretaceous nodules appeared to him to be the remains of old blood-clots, which had originally formed in the fallopian tubes."

Are there no signs of the fatal conflict in this case?

At the same meeting Dr. Harris, of Philadelphia, read a paper in which he states: "I met in New York a woman, of twenty-six years of age, having *her heart so frightfully diseased* that she was not only a great sufferer, but looked upon by physicians as a living curiosity, whose affection resulted from rheumatic inflammation *produced by her having bathed her genitals in cold water at the age of thirteen*, under the impression that the menstrual flow was a bleeding from some accidental injury, her mother having never warned her of the event, nor instructed her as to what she should do upon its appearance."

Might not a careful investigation find marks of the *conflict* here?

In St. George's Hospital, London,* was admitted May 30th, 1872, J. C., a girl aged eighteen; "her family was healthy; she was single and *had never seen any catamenial discharge;* but for

* Lancet, New York, November, 1872. p. 593.

three months before admission she had, from time to time, suffered pain at the lower part of the back and between the shoulders. During these attacks of pain she had *bleeding from the nose and gums, which lasted about a week, and then ceased, returning again after the interval of one month.* For two or three weeks before she came to the hospital she had great irritability of her skin, to relieve which she had recourse to scratching, but this gave rise to immediate bruising of the parts. For four months past she had complained of pain in the left side, accompanied with difficulty of breathing, cough and spitting of blood. *She had never had rheumatic fever;* but about four years ago she suffered from chorea.

"On admission she was very anæmic, the lips and conjunctiva being almost bloodless. She suffered from shortness of breath, and had frequent bleedings from the nose, mouth and skin. She said she had never menstruated. There were hemorrhagic spots on the tongue, inside the lips, and on the gums. Some of the spots on the tongue were as large as half a split pea, and the tip was so covered with ecchymoses that it had the resemblance of a strawberry. The lips were cracked, and on the inner side were numerous ecchymosed spots. The surface of the chest was more or less marked with these hemorrhages, but here some of the spots could be picked off; at the places where scratching had been practiced there were distinct bruises. On the legs and thighs the spots had more the character of the hemorrhages seen in purpura. In many places the blood seemed to have actually exuded from the skin, as they could readily be lifted off; but there was no evidence that mechanical means had been employed to produce them. For four or five days she had suffered from epistaxis. On examining the chest, a loud mitral murmur, most marked at the apex, was heard, the heart's action being very irregular and rapid. The lungs were resonant, and air freely entered; but the breathing was rapid and labored, even after slight exertion. There was troublesome cough, and occasionally the patient spat blood. There was no vaginal orifice; the small cavity representing the canal of the vagina ended in a cul-de-sac, and was not deep enough to hold a teaspoonful of fluid. The urethra was in the middle of this cavity. The labia majora were well-formed, but small, and there was an ordinary amount of pubic hair. The space between the rectum and the urethra measured about half an inch. On passing the finger into the rectum, no uterus could be discovered; and when a catheter was introduced into the bladder, it could be distinctly felt through the anterior wall of the rectum. Numerous ecchymoses were present on the inner side of the labia majora. * * * The patient continued to improve until 11th June, when the breathing became much embarrassed, and accompanied with severe palpitation of the heart, cough and spitting of blood; death taking place at 3 P. M., consciousness remaining till the last.

"Autopsy: Body *well nourished;* limbs and trunk covered with ecchymoses. Mammæ fairly developed, but nipples small. On opening the thorax the pleuræ were found to be spotted with ecchymoses. The lungs were œdematous, and *gorged with blood.* The pericardial cavity contained a small quantity of light, red fluid, but the walls were dotted with hemorrhagic spots, especially the visceral wall. The endocardium at the upper part of the left ventricle was thickened and opaque. *The aortic valves were thick, puckered and inefficient, the mitral valve thickened, and so contracted that the orifice would only admit the tip of the little finger. The muscular walls of the right ventricle and left auricle much hypertrophied.* The liver, spleen, and kidneys did not present any abnormal appearance. *The ovaries were very well developed and congested, and contained a recent false corpus luteum.* The uterus was absent, (evidently congenitally,) only a small nodule of fibrous tissue being found in the folds of the peritonæum, between the rectum and the bladder."

Surely, we have the mortal wounds of the recent conflict here.

At the annual session of 1872, in Columbia, of the South Carolina State Medical Association,* Dr. T. G. Simmons, of Charleston, relates the history of a "Case of Atresia Vaginæ; Death from Tetanus at Menstrual Period, with Account of Post-Mortem Appearances." M. E., a negress, primipara, was delivered by craniotomy and evisceration on the 5th of May, 1869. Atresia vaginæ followed, and in December of the same year an unsuccessful attempt was made to reëstablish the vaginal canal on account of her suffering with symptoms of retained menstrual discharge. Dr. Simmons says: "The patient continued under

* Transactions South Carolina Medical Association, 1872. p. 56.

my care *suffering intensely* at each recurring menstrual period, but never showing any appearance of an accemulation. I resorted to various anodyne and anti-spasmodic agents for her relief." November 19th, 1870, another attempt was fruitlessly made to remedy the difficulty. On the 14th July, 1871, says Dr. Simmons, "I saw her, and found her suffering *intensely* with pains in back and limbs, also some rigidity in recti-abdominales, pains and rigidity in masseters. These I deemed to be hysterical phenomena. * * * The next morning, found she had passed an uneasy night, but had slept at intervals. The tetanic symptoms well marked, but the paroxysms not very violent or frequent. Patient does not present any wound, nor is she aware of any. * * * (27th July.) I saw her again in the evening; found her much worse; the remedies now seemed of no avail to relieve her of the pain or relax the severity of the spasms. Jaws tightly locked, but still able to swallow fluids; opisthotonos. She remained in this state of fearful suffering until the morning of the 30th, when death closed the *dreadful tragedy*, sixteen days from its commencement. * * * Autopsy, fourteen hours after death: Some degree of emaciation; rigor mortis well established; abdominal organs presented a healthy appearance; the uterus small and bound down, anteflexed and laterally displaced to the left by strong bands of cicatrical tissue; the left ovary prolapsed and the fallopian tube adherent to the uterus, extending down over that organ; and the fimbriated extremity firmly attached to and opening into the vagina on left side, about one and a half inches from the uterus, and at point of opening made at time of operation. The vagina occluded by dense fibrous structure for one and a half inches, the cicatrix being continuous in structure with uterus and inseparable from it. The right ovary and fallopian tube presented nothing worthy of note. There were no recent corpora-lutea *apparent* on either ovary. * * * The seat of attachment of the fallopian tube at its entrance into the vagina was stained dark-red from recent sanguineous discharge through it. * * * *The left ovary was bound down to the uterus by broad bands of adhesions;* length of uterus two and a quarter inches; width at widest part one and three quarter inches. The uterine canal was obliterated, except at either corona; no trace of cavity or os to be found.

The fallopian tubes, on both sides, obliterated from uterus to ovary, but patent from fimbriæ to ovary. The fimbriated extremity of right fallopian tube presented no appearance of recent discharge, nor were there any appearances of hemotocele to be found. The pelvis was contracted antero-posteriorly to two and a half inches."

Dr. T. R. Brown, of Baltimore, reports in the October (1872) number of the *American Journal of Medical Science*, p. 575, a "Case of Abnormal Structure of the Female Genital Organs." "I was called some three months ago to a lady, nineteen years of age. * * * Four days previous to the patient's death she had quite a profuse bleeding at the nose, lasting about forty-eight hours, and concluding with a sort of nasal catarrh, which, taken with the facts that she had never menstruated, and that the nose bleedings were frequent in their recurrence, induced an examination, post-mortem, of the internal organs of generation. * * *

"The vulva was natural in formation and appearance, with the mons veneries and external surface of labia majora well covered with hair; no clitoris could be perceived. The vagina, which was a simple cul-de-sac, about two inches long, was dissected out without encountering the crura clitoridis. It was destitute of rugæ, hymen, caruncular myrtiforms, and had no communication with the os uteri. The bladder and rectum were firmly adherent to each other, instead of being separated by an uterus, for which we hunted in vain. The bond of adhesion between the bladder and rectum was the broad ligament, occupying its usual position, of a crescent shape; and imbedded in *either* horn of this crescent, near the summit, about one and a half inches internal to and on a line with the iliac fossæ, was a nodular body, dense in structure, of the size of an apricot kernel, to which were attached a perfect ovary, fallopian tube, and round ligament. The parts adjacent to the ovaries were *greatly congested, evidently connected with a recent ovulation;* and an incision into one of the ovaries showed several corpora lutea, with their corresponding cicatrices, on the outer surface.

"I am of opinion that the nodular bodies referred to were what would correspond to the superior corona of the uterus, and the non-striated muscular fibre, found by Dr. Tiffany in a small section, confirms my impression of its being uterine tissue. The mammæ were unusually well developed, and the symmetry of the patient's figure well illustrated the vigor of her previous health, and her powers of endurance in sickness."

In a private letter, Dr. Brown writes me: "Her *physique* was a model of anatomical symmetry and female beauty; breasts well developed, as were also the external organs of generation. Owing to the condition of her intellectual faculties, her previous history could not be accurately obtained. Enough, however, was found out to satisfy me that there was an abnormality as to her menstrual sickness, which had been taking place for some time in the form of epistaxis, two of which hemorrhages ocurred during the time of my treatment of the case, with the usual interval of about four weeks, at which times all the symptoms of the disease (afterwards diagnosed to be cerebal meningitis) were more pronounced. She voided daily a gallon or gallon and a half of normal urine; later on, involuntarily drenching her bed and clothing several times during the day. These, and other symptoms, simply corroborative as to the character of the malady, terminated in profound coma, which continued up to her death. As I have said before, I regard this case as being cerebral meningitis, which, very probably, was associated with some cerebritis, and that *the disease was the result of suppressed uterine menstruation* acting upon a mind already much concerned and excited upon the subject of some possible defect existing in the organization of her generative apparatus, together with the relation of such defect to her matrimonial prospects."

Dr. Byford* says: "The debility, the imperfect or perverted hæmatosis or the nervous energy, seldom becomes so great as to be the *immediate* cause of death. This, however, sometimes does occur, and *we should indulge a false security to suppose that our patient could not thus die.* I think I have seen more than one instance of death thus resulting. * * * As very correctly stated by Dr. Bennett, *such an unnatural condition of the nervous system and blood is engendered by the disease as to destroy the capacity of the patient to resist or ward off the attacks of the acute diseases to which she may be exposed, or the chronic ones for which she may inherit a strong predisposition.*"

What say you then, my brethren, may a woman *die* because of unrelieved menstrual molimen?

* Medical and Surgical Treatment of Women. p. 174.

I shall not detain you with any authorities or argument to show that this condition is *destructive of human health.* I have shown that it may be destructive of life; *ergo,* it may be destructive of health. But I appeal, confidently, to the individual observation of each one of you for the proofs of this proposition.

*It is destructive of human reason.* I shall detain you with but a single authority upon this point, that of Prof. Fordyce Barker* who says: "Now, appreciating these phenomena, which are physiological in most women, you will be prepared to believe that a pathological condition of these organs, and an *impairment or arrest of their functions,* may be a cause of great disturbance of the circulating and nervous systems, and may result in absolute derangement of the cerebral functions. Although these are scarcely referred to by the systematic writers on female diseases, yet every one who has had much clinical experience in these diseases must have seen their results more or less frequently. Every insane hospital probably contains more or less of such cases, and the special writers on mental diseases furnish numerous illustrations of this fact."

"Of cases of insanity which were induced by amenorrhœa, (I say so because the cure of the amenorrhœa was followed by an entire disappearance of the insanity,) I have seen two," which he goes on to relate, and also the history of a third case which ended fatally. "The autopsy absolutely revealed nothing to explain the cause of death. The ovaries were normal as to size and structure, but *somewhat congested, and on one was the most marked and beautiful specimen of recently ruptured Graafian vesicle* that I have ever seen, while the uterus was less than half the size of the virgin uterus. Its cavity did not contain one drop of blood, but its lining membrane had numerous points of ecchymosis."

It is also *destructive of human happiness.* Think you, my brother, when you have felt the pulse, eased the bodily pain, cooled the burning fever, arrested the paroxysm, that you have done *all* that our noble profession exacts at your hands? Have you nothing to do as to the precious pearl which the human casket is but formed to enclose? Is the heart and the soul of your patient no concern of yours? We can not forget that some

*Journal of the Gynæcological Society, May, 1872.

of the brilliant exploits of surgery have chiefly for their object the *happiness* of mankind, in the removal of unsightly deformities. Have you no care for woman's happiness? Within the boundaries of an adjoining State, but a few years ago, there lived and labored an obscure practitioner of our beneficent art, eking out a livelihood for himself and family, his fertile brain and kindly heart the solace and hope of his humble patrons. *Now* he is gone forth to receive the homage of the medical world, and to inscribe his name so high upon the pinnacle of fame that remote generations of man may see and read it.

Do you ask me why all this homage—why all these honors? I answer you, *his attentive ear was open to the cry of human misery.* He entered the hut of a lonely prisoner, an outcast from society, an object of disgust and abhorrence to her own family and friends; nay more, an object of loathing to herself, *because of the stench of her incontinent urine.* She was not sick, she needed no medicine, and yet his ear was open to her cry. In meditation deep, his busy brain scintillating with brilliant thought, he stooped him down, as did his Lord and Master, and with his finger wrote upon the ground. And lifting himself up he said unto her, "Woman where are those thine accusers? hath no man condemned thee?' She said, 'No man, Lord;' and he said unto her, 'Neither do I condemn thee.'" And, in imitation of his Divine Archetype, he removed her shame, and taking her by the hand he led her forth, and restored her to the world and to *happiness;* and to-day the voice of woman, reverberating from peak to peak, echoes the name of Sims all around the habitable globe!

Again, it is *incurable by the recognized resources of the art.* Allow me to quote you a few authorities upon this point. Prof. Thomas* says: "The prognosis of chronic inflammation of the uterine body is *always grave* with reference to cure. Even if the case is not of very serious character, and has lasted only a short time, the possibility of rapid recovery is doubtful, while, if it has continued for a number of years, *it will often prove incurable.* * * * In most cases a certain amount of amelioration may be effected even when they are of long standing; in a certain number treated early, cure may unquestionably be ac-

* Diseases of Women. p. 255.

complished ; while in a great many *nothing whatever, either in the way of cure or relief*, can be obtained, and the patient, after passing from physician to physician, settles down into a careful mode of life, resolved to cease treatment and bear as best she may an evil which she has learned to regard as *incurable*."

Prof. Thomas also goes on to remark that the prognosis is unfavorable "when the case is of long standing; the nervous system is involved; patient *not near the menopause*."

Speaking to the same point, Scanzoni* says : "Unfortunately, the favorable time for the radical cure is ordinarily past, and we may esteem ourselves fortunate if we can but moderate somewhat the hypersecretion of the uterine mucous membrane, and moderate its consequences. As for ourselves, we do not remember *a single case* where we have been able completely to cure an abundant uterine leucorrhœa of several years' standing. We have already said that the severer forms of this malady may become *dangerous to the general organism;* and many women, whom we have been called upon to treat, had to attribute to the neglect of the disease a bodily and mental debility, which they would keep for the rest of their days, or hysterical attacks, which *deprive them of all enjoyment of life*."

Dr. Byford† says : "We should temperately encourage our patient, if we can conscientiously do so, and if our judgment will not allow us to do so, we should express, temperately and cautiously, an unfavorable prognosis ; and *hope should never be extinguished until the patient is moribund.* Too many good reasons will suggest themselves for the last course to require any argument in support of it. What I have said of a guarded prognosis, and the necessity of not giving a sweeping and absolute opinion, seems to me peculiarly applicable to the disease of which I am now treating. Physicians have not all been convinced of the propriety of treating uterine diseases with the speculum ; a large number are entirely, and conscientiously, opposed to it. They are made so, undoubtedly, by the failure of local treatment to fulfill the hope originated by its most ardent advocates. *It does not do what they are told it will do;* it certainly does not in all cases. The only grave error, I think, committed by that benefactor of mankind, Dr. Bennett, in his

* Diseases of Women. p. 202.

† Medical and Surgical Treatment of Women. p. 172.

work on the unimpregnated uterus, is that his book leads his readers to believe that he scarcely, if ever, fails to cure his cases. This is the impression made upon most physicians who read his book. However true it may be, with reference to the practice of so able a master, I think it would be an unjustifiable expectation on the part of the profession at large. From what I have heard and read of the opposition of medical men to local treatment in uterine disease, I think this unrealized expectation of success from local treatment, is one of the main causes of it. Upon trial, medical practitioners become disappointed with the results as they were led to expect them, and abandon the plan as a failure. While I cannot coincide with Dr. Bennett as to the almost universal success of local treatment for uterine inflammation, I am of the opinion that it is greatly superior to any other with which I am acquainted. Prognosis must depend for its reliability, to some extent at least, upon a correct and complete diagnosis of the whole condition of the patient."

*Are cases of chronic corporeal endometritis, accompanied by amenorrhœa of fifteen years' duration, easily cured? Are they generally curable? Are they curable at all?*

Does somebody object that the *ovaries are normal*, and we must not sacrifice a healthy organ for the sake of a diseased one? Is a part greater than the whole? Is the integrity of one organ superior to that of the whole body? Must the erratic function of the ovary be allowed to subvert the functions of the entire organism? But this principle has been settled upon the highest possible authority, and for more than eighteen centuries. "Wherefore if thy hand or thy foot offend thee, cut them off, and cast them from thee; it is better for thee to enter into life halt or maimed, rather than having two hands or two feet, to be cast into everlasting fire."

Is it objected that normal ovariotomy is a *reproach to gynæcology?* I answer in the language of an honored teacher, Prof. S. D. Gross, of Philadelphia: "As long as the human body is liable to accidents, and as long as nature is incapable of arresting, by her own efforts, the various morbid processes which she herself institutes, so long will practitioners be compelled to invoke the aid, and, I may add, the blessings of operative surgery. Is it a disgrace to amputate a leg for a mortification of the foot, to extirpate a testicle that has been destroyed by cystic disease, to divide the stricture in strangulation of the bowel, to extract a stone from the bladder? * * Surely no one will doubt that in these, and a hundred other instances, our object can be attained only by an operation. Medicine, under such circumstances, however judiciously administered, is not only utterly futile, but is always ready to avail itself of the aid of surgery. Its empire is temporarily suspended, and it only resumes its legitimate function after the use of the knife."

A similar charge has been brought against an operation of which Dr. LeFebre, of Belgium, says: "Ovariotomy is a *precious conquest* for modern surgery, and must not be abandoned." And Sir Wm. Ferguson, nearing the close of a long life made brilliant by his surgical researches and practice, standing upon the very pinnacle of British surgery, his whitened locks encircling an honored brow, says of it: "The proceeding is a well-established fact, *which ranks among the best and safest efforts in surgery to save human life.*"

Do I hear some little soul whisper, "An innocent woman suffers that a doctor may gain notoriety?" There are, I fear, even in our own professional household, narrow minds which, like the frightened ostrich of the plain, hide their diminutive heads beneath a scanty covering of envy and prejudice, where they can see nothing but their own gaudy plumage, and vainly imagine themselves safe from the shafts of Achilles; narrow minds, whose obscured vision finds little within the great temple of science, in which we worship, besides the gilded horns of the altar and the glittering cornice and panel work; narrow minds, whose meagre ken is incapable of appreciating the nobler conscientiousness of duty discharged for duty's sake.

In retrospecting the ground we have gone over, you perceive I have proposed for your acceptation a new operation in surgery, which I believe to be original with myself in its conception, original in its elaboration, and original in its successful execution. I have related to you the history of the case up to the present time. I have endeavored to show you that the change of life was a reasonable remedy for the morbid conditions present in the case; that it was reasonable to expect that the removal of the ovaries would determine the change of life. I have asked you to hold fast to your faith in the ovular theory of menstruation, notwithstanding some anomalous results of double ovariotomy.

While admitting, for myself, the danger to life from the execution of normal ovariotomy, I have asked you to admit, upon the other hand, that this danger is not great, and that it is not out of proportion either to the severity of the malady upon the one hand, or the magnitude of the results upon the other. I have admitted that sterility necessarily followed the operation, but I have also asked you to admit that, in ali human probability, it also preceded the operation, and is not to be charged as one of its consequences.

I have denied that the operation involves loss of the aphrodisiac sense. I have shown you that, in numerous instances, after double ovariotomy, it was retained in full vigor; and I now challenge you to show one instance of its loss.

I have admitted the exceptional loss of the feminine graces, and I ask you also to admit that such results, after double ovariotomy, are exceptional, *very exceptional*, and do not invalidate the general rule that there is no such loss. I have endeavored to satisfy you that an unrelieved menstrual molimen is destructive to life; that it is eminently destructive of health and happiness; and that the condition is often incurable by the recognized resources of our art.

And now it but but remains to inquire, WHAT IS OUR DUTY?

Heartily do I adopt and commend to you, my brethren, the sentiment of Dr. Charles West,* an opponent of ovariotomy: "One remark I can not refrain from making on the grievous injury that is done both to the advance of medical knowledge and to the standing of our profession with the public, by the practice of treating some of these questions as though they were *questions of moral right or wrong.* It would seem, from what has sometimes been said on the subject, almost as if ovariotomy could not be defended save from some sinister end, nor its expediency be doubted except from a moral obliquity rendered excusable only by hopeless dullness. Belief in each other's integrity of purpose seems to me essential to our eliciting truth by discussion; and I see no reason why I am to suspect another of being less mindful of our common duty to humanity because

* Lectures on Diseases of Women. p. 441.

he tries to relieve suffering, or to prolong life, by some means in which I have not the same confidence.

"Your duty and mine is, not to sit down in apathetic indifference, doing nothing, trying nothing, for a patient's cure, because her disease is one which hitherto has proved almost invariably mortal; but rather, patiently, carefully, with much mistrust of our own powers, much watchful scrutiny of our own motives, to apply ourselves to the trial of *every means* by which suffering may be mitigated or life prolonged. To this our common humanity prompts, our obligations as medical men compel us. It is to misinterpret both, very grievously, if we not merely content ourselves with doing nothing, but take shelter under noisy censure of the conduct, and uncharitable construction of the motives, of those who read their duty differently."

In the rapid strides which gynæcology has taken of late years, we may indulge the hope that the field for normal ovariotomy will become still narrower than now, but in the meantime what are we to do for these hopeless invalids? Are they to be allowed to suffer on year after year without hope, except in the interposition of nature at the climacteric?

Suppose a patient of middle age come to you with a stone in his bladder, undermining his health and destroying his happiness; will you look at your *life-tables* and say to him: "Sir, you must be patient; you must drink freely of the diuretic teas and of the mineral waters; perchance they may diminish somewhat the size of your stone; and if they do not do this, they will, at all events, render you more comfortable, and enable you to bear sufferings which you can not remove by medicines. Nothing but the knife can cure you, and really I can not *cut* you; you might die, and besides it might render you impotent, so that you could not become the father of a family. Such things *have* happened, and who knows but it might happen to you? See here!—here is the life-table of general averages; your reasonable expectation is ten, fifteen, perchance twenty, years; then nature, herself, will relieve you of all your sufferings. Sir, you must really be patient and hope for the best."

Do you thus read your duty? Is it any more dangerous to life than an unrelieved menstrual nisus; any more destructive to human health; to human happiness?

But there is a class of cases of which West says, "They are completely beyond the reach of remedy;" of which Hodge says, "Disappointment must often ensue, as the causes of amenorrhœa are frequently immovable;" of which Byford says, "In all cases of absence of the ovaria or uterus, we could not expect to do good by any treatment;" of which Thomas says, "If the uterus be absent, all that can be done will be to abstract a sufficient amount of blood from the arm, by venesection if necessary, to relieve the urgent symptoms attending each epoch." Does our duty to these resolve itself into a mere meditation upon the wonderful powers of endurance with which our Creator has so miraculously endowed woman? And what of those who, though having a uterus, are yet partakers alike with the others in the same sufferings, the same despair, the same death?

Is it enough that we follow the injunction of old Thomas Rainalde, as laid down in his Woman's Book, "To instruct and comfort the party, not only refreshing her with good meat and drink, but also with *sweet words*, giving her good hope of a final deliverance, encouraging and *enstomaching* her to patience and tolerance, etc." Shall we give her some doses of camphor, of musk, of valerian, of assafœtida? "Things that to hear them told, have made me tremble;" things of which she has already taken gallons, and for years; things, the very name of which is a stench in her nostrils to this day! Shall we take some ounces of blood from the arm with our thumb lancet? Nature has been bleeding her scores and scores of times, and all to no purpose, save to ward off for the time impending death.

Am I told of the restorative powers of the mountain air, of cheerful society, of travel, of the mineral waters? As well descant to the famishing ditcher in the bogs of Ireland of the toothsome delicacies of Delmonico's or the Trois Freres; as well remind the hapless traveler, with mangled limb crushing beneath the ponderous train, of the soothing influences of music, and of the gentle evening zephyr.

In all this large and learned and grave assembly, am I alone in my experience, when, after years of study, of patient investigation and earnest labor, I am forced to say to my discouraged patient: "It is enough; I have faithfully studied and labored your case; I have exhausted the materia medica; I have tried every expedient; for you there is no balm in Gilead; henceforth you can only look to the change of life for remedy." Can it be that after years of such sad experience, I have come up here to learn, at last, a ready solution for so difficult a problem in the old and familiar resources of our art? Happy indeed would be the day; thrice happy the man who should thus return to his suffering, hopeless patient, joyfully shouting—Eureka! Eureka!

Go with me to the chamber of a solitary invalid, worn down to a shadow by her long years of painful suffering, heart-sick with hope deferred. See her as she sits in her solitude of despair—the winding-sheet of her own living sepulture gathered about her—anxiously peering into the dark, unknown future, and impatiently waiting to catch, if possible, a glimpse of the fleet-footed, ghastly messenger, bearing the sand-glass and the scythe —her last, her only friend!

Go with me to the family fireside; see her now around the cheerful hearthstone, the sunny smile of contentment and hope replacing the gloomy clouds of despair. See with what interest and zeal she engages in the duties and the pleasures fitting to her age and sex. Already are the deep furrows and angular prominences being smoothed and rounded by adipose deposits; already is she attaining somewhat of the beauty and grace which the promise of her girlhood had foreshadowed. She feels that she is now anchored in a secure harbor, from which she can look back with composure upon the many fearful storms through which she has passed, and which she may no longer dread. And though she can not claim for the future the rewards of maternity, she is thankful that she escapes its perils and its cares. Failing in other objects of her womanly love and devotion, she finds in her church, and in the great cause of humanity, ample scope for all her heart's desires.

Look upon this picture, and then upon this, and tell me, my brethren, which do you elect?

Do I address some who, though rejecting my proposal for remedy, have nothing effective to offer instead; some who, "laden with years, furrowed with the routine of long experience, to whom a certain unvarying regimen has become as a part of their nature, still entertain the belief that the school in which they were taught, and the maxims therein imbibed, embody the principles of a system—the type of perfection—not to be disturbed by additions and improvements, so denominated, and from which nothing can be safely or advantageously removed?" To such I would respectfully say, in the language of England's great dramatist:

"Tell me not, Friar, that thou hearest of this,
Unless thou tell me *how I may prevent it.*
If in thy wisdom, thou canst give me help,
Do thou but call my resolution *wise,*
And with *this knife* I'll help it *presently.*
* * * * * *
Therefore, out of thy long experienced time,
Give me some present counsel; or behold!
'Twixt my extremes and me this bloody knife
Shall play the umpire; arbitrating that
Which the commission of thy years and art
Could to no issue of true honor bring.
Be not *so long to speak;* I long to die,
If what thou speak'st speak not of *remedy.*"

What say you then, my brethren? If we believe in our hearts that the change of life will cure these direful maladies, ought we still to content ourselves with sweet and honeyed words, encouraging and enstomaching to patience and resignation? Ought we not rather to bring down the clenched fist in manly determination of purpose, exclaiming, by the eternal powers *let the change of life come*—Now! NOW!

